# Autosplenectomy in a Patient with Paroxysmal Nocturnal Hemoglobinuria (PNH)

**DOI:** 10.1155/2019/3146965

**Published:** 2019-02-12

**Authors:** Ethan Burns, Kartik Anand, Gonzalo Acosta, Malcolm Irani, Betty Chung, Abhishek Maiti, Ibrahim Ibrahim, Lawrence Rice

**Affiliations:** ^1^Houston Methodist Hospital, Department of Medicine, 6550 Fannin St, Houston, TX 77030, USA; ^2^Houston Methodist Hospital, Department of Pathology and Genomic Medicine, 6550 Fannin St, Houston, TX 77030, USA; ^3^The University of Texas MD Anderson Cancer Center, Division of Cancer Medicine, 1515 Holcombe Blvd, Houston, Texas 77030, USA; ^4^University of Texas Southwestern, Department of Internal Medicine, Division of Hematology/Oncology, 5323 Harry Hines Blvd, Dallas, TX 75390, USA

## Abstract

Autosplenectomy (AS) is a known complication of diseases such as sickle cell anemia, celiac disease, and inflammatory bowel disease. We report the first known case of AS due to paroxysmal nocturnal hemoglobinuria (PNH). A 24-year-old Caucasian male had evidence of hemolytic anemia at the age of 14 and was diagnosed with PNH at the age of 16. He had recurrent episodes of sepsis due to dialysis line infections from poor hygiene, and blood cultures had been positive for multiple organisms including *Staphylococcus aureus*, *Enterococcus faecalis*, and *Streptococcus pneumoniae*. The patient's peripheral blood smears since the age of 14 years demonstrated Howell–Jolly bodies in conjunction with thrombocytopenia and hemolytic anemia, but abdominal ultrasonography reported a normal appearing spleen. The patient presented with septicemia two years after starting eculizumab, and his peripheral blood smear showed extensive Howell–Jolly bodies, Pappenheimer bodies, acanthocytes, and target cells. Splenic ultrasonography demonstrated an atrophic spleen with multifocal scarring, and absent splenic uptake of liver-spleen scintigraphy, consistent with AS. Clinicians should remain vigilant of the potential sequelae of PNH and consider the possibility of the development of AS.

## 1. Introduction

PNH is an acquired hemolytic disease involving the hematopoietic progenitor cells of the bone marrow. The incidence and prevalence of PNH is 0.13/100,000 and 1.59/100,000 person/year, respectively [1]. Symptoms vary and arise secondarily to ongoing hemolytic anemia and thrombophilia and may include fatigue, urine discoloration, and pain. The only FDA approved therapy is eculizumab, a humanized monoclonal antibody that prevents the cytolytic and proinflammatory effects of the terminal complement system by acting against the C5 component of the complement cascade [2].

Hemolytic diseases can lead to repeated splenic infarcts, resulting in splenic atrophy and eventual loss of function. This can be histopathologically correlated with the presence of basophilic nuclear remnants in circulating erythrocytes known as Howell–Jolly bodies. In general, infiltrative hematologic diseases and thromboembolic diseases may predispose the spleen to infarction [3]. The following case illustrates the first known report of AS attributed to PNH.

## 2. Case

A 24-year-old Caucasian male with a history that includes Addison's disease, developmental delay, hypogammaglobulinemia, PNH complicated by lower extremity thrombosis, minimal change disease (MCD), and end-stage renal disease (ESRD) presented with headaches, fevers, and neck stiffness for several days after a tunneled dialysis catheter was placed for hemodialysis.

The patient was diagnosed with Addison's disease at the age of 14 years following a workup for ongoing fatigue. At that time, he was also found to have macrocytic anemia (hemoglobin 9.3 G/dL, hematocrit 26.9 G/dL, and mean cell volume 114 FL), thrombocytopenia (platelets 16,000 U/L), elevated lactate dehydrogenase (1261 U/L), and reticulocytosis (3.6%). His direct antiglobulin test, platelet antibodies, and HIV antibodies were negative, and the ADAMTS13 activity level was normal. Hemoglobin electrophoresis was not suggestive of hemoglobinopathy. His peripheral blood smear reported anisopoikilocytosis, mild to moderate schistocytes, and Howell–Jolly bodies. Splenic ultrasound with Doppler studies were normal. Slightly increased osmotic fragility was present, and bone marrow biopsy reported a hypercellular marrow with predominance of erythroid precursors. Further evaluation including bone marrow biopsies, cytogenetic analysis, and autoimmune panels failed to confirm a cause for this patient's hemolytic anemia. After returning to the clinic with progressive fatigue and hematuria, the diagnosis of PNH was made when flow cytometry indicated PNH clonal populations in 38% of red blood cells (RBCs), 68% granulocytes, and 71% monocytes. Since the patient's anemia was transfusion independent and without evidence of thromboembolic disease, treatment with eculizumab was not offered until two years after the confirmed diagnosis which the patient refused. His renal function declined over time, and initial renal biopsies reported hemosiderosis due to PNH. His renal function continued to worsen over the next three years, and he eventually developed significant proteinuria (20 g/24 h) prompting repeat kidney biopsy. The results indicated significant hemosiderosis due to PNH as well as effacement of podocyte foot processes consistent with MCD. He was treated with prednisone and later rituximab for MCD. Rituximab was later discontinued after the patient developed new onset hypogammaglobulinemia after starting rituximab. His proteinuria resolved with continued steroid therapy, but his renal function did not improve, requiring initiation of hemodialysis. After the diagnosis of ESRD, the patient was agreeable to eculizumab and started therapy. The patient's clinical course was further complicated by recurrent catheter-associated infection.

Over the past two years, the patient has been treated for frequent episodes of sepsis due to coagulase-negative *Staphylococcus aureus* bacteremia, methicillin-sensitive and resistant *Staphylococcus aureus* bacteremia, *Enterococcus faecalis* meningitis, and *Streptococcus pneumoniae* bacteremia and pneumonia. It was thought that the patient's recurrent infections were from poor tunneled dialysis catheter hygiene and maintenance.

**Figure 1:**
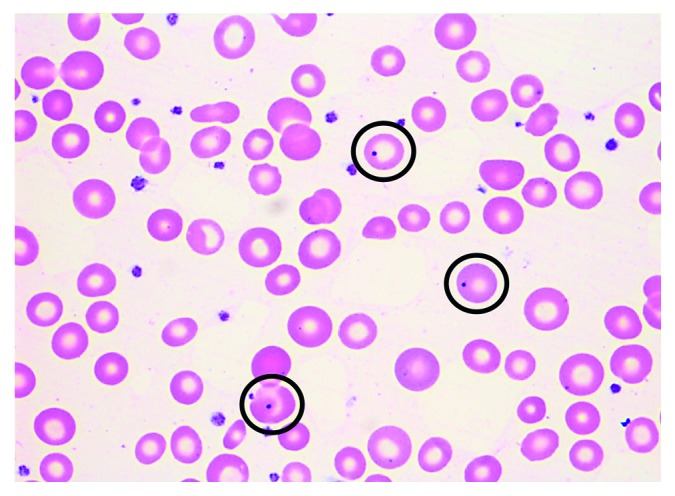
Peripheral blood smear (1000x oil, magnification): normocytic normochromic anemia with mild anisopoikilocytosis and three erythrocytes (black circles) with Howell–Jolly bodies (nuclear remnants) and adequate numbers of appropriately granulated platelets.

**Figure 2:**
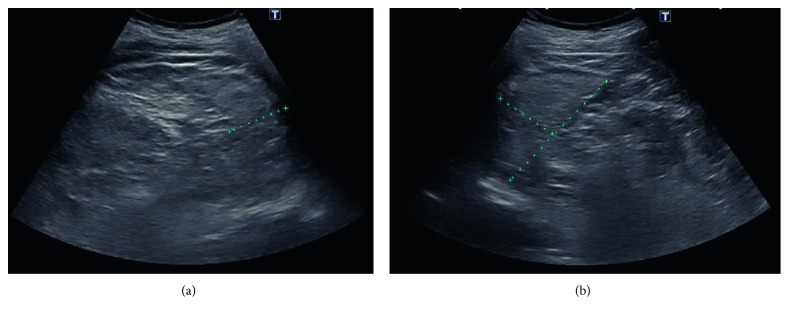
Splenic ultrasound. (a) Transverse dimension: 3.3 centimeters. (b) Long axis: 7.9 centimeters and anteroposterior: 3.9 centimeters. Atrophic spleen suggestive of multiple repeated episodes of infarction.

**Figure 3:**
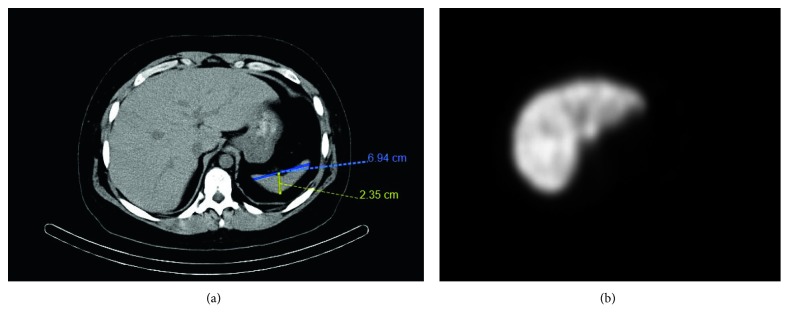
(a) CT showing atrophic spleen without the presence of calcification. (b) Liver-splenic scintigraphy demonstrating hepatic uptake of radioactive ^99m^Tc-labelled colloid but absent splenic uptake.

On the current admission, peripheral blood smear demonstrated the presence of numerous Howell–Jolly bodies (Figure 1), Pappenheimer bodies, acanthocytes, and target cells. Splenic sonogram now showed multifocal scarring and splenic atrophy, measuring 7.9 × 3.9 × 3.3 cm, suggestive of recurrent infarctions (Figure 2). Liver-splenic scintigraphy with ^99m^Tc-labelled colloid did not show splenic uptake, consistent with AS (Figure 3). The patient continued to have recurrent episodes of staphylococcus bacteremia, leading to tunneled dialysis catheter removal and transition to peritoneal dialysis.

## 3. Discussion

PNH is caused by an acquired somatic mutation in the X-linked phosphatidylinositol glycan complementation class-A (*PIG-A*) gene that results in an absence of glycosylphosphatidylinositol linked surface anchored proteins on hematopoietic stem cells [4]. This leads to an absence of the decay accelerating factor (CD55) and membrane attack complex-inhibitory protein (CD59) on the surface of hematopoietic cells. Most human cells, including platelets and white blood cells, express surface CD55, CD59, and membrane cofactor protein (CD46), another inhibitor of complement-mediated effects [4]. Erythrocytes lack CD46 [4], and in the presence of a *PIG-A* mutation, circulating CD55 and CD59 deficient red blood cells cannot prevent complement binding, leading to complement-mediated intravascular destruction. Chronic hemolysis is due to unregulated activation of the alternative pathway of the complement system [5]. The alternative, lectin, and classical complement pathways converge at C3 activation. C3 spontaneously hydrolyzes and leads to the formation of C3 convertase, which in turn activates C3, C5, and the membrane attack complex (MAC) [5]. The presence of CD59 interrupts the formation of a fully functional MAC via inhibition of C9 polymerization; in the absence of CD59, unregulated formation of MAC leads to chronic intravascular hemolysis [5]. Transient decreases in pH or stress imposed on the body by surgery, infection, or inflammation may enhance complement-mediated effects, which may perpetuate brisk intravascular hemolysis and precipitate recurrent hypercoagulable events [5].

In sickle cell anemia (SCA), the spleen is one of the first organs injured as the disease manifests [6]. Though it may be clinically silent, evidence of AS with Howell–Jolly bodies is often present within the first 12 months of life, with complete loss of splenic function by 5 years [6]. The mechanism of splenic destruction is related to a combination of upregulation of RBC adhesion molecules, diminished RBC deformability causing occlusion of splenic microcirculation, and recurrent vaso-occlusive episodes leading to progressive fibrosis, atrophy, and eventual AS [6]. This case demonstrated evidence of splenic damage on peripheral blood smear during the patient's initial presentation of hemolytic anemia, indicating that splenic damage may have started to occur early in PNH onset and suggesting a relationship between this patient's PNH and AS. Over time, it is possible that treatment delay and recurrent septicemia-induced PNH exacerbations led to repeated splenic ischemic infarcts, eventually resulting in AS.

The mechanism of PNH-induced AS is unknown, but hypercoagulability may be involved. In PNH, thromboembolism can occur at any site including the intra-abdominal vasculature and is attributable to 40–67% of deaths [7]. Approximately 29–44% of patients will have thromboembolic disease over the course of PNH [7], and patients with a granulocyte colony factor count of >50% have a 34.5% 10-year incidence of thrombosis [8]. Although less common, arterial thrombosis may also occur, usually in the cerebral or coronary circulation [9, 10]. Arterial complications including ischemic strokes and myocardial infarction have occasionally been described as the initial manifestations of PNH [9, 10].

PNH-induced hypercoagulability occurs via a myriad of proposed mechanisms. During hemolysis, a decrease in nitric oxide (NO) production and an increase in circulating cell-free hemoglobin-mediated NO scavenging results in enhanced endothelial molecule expression and cGMP mediated platelet activation [11–15]. In addition, the tissue factor (CD142) is abnormally expressed in hemolytic anemias including PNH and is thought to contribute to hypercoagulability [16, 17]. Furthermore, procoagulant phospholipid microparticles released during episodes of hemolysis may also contain elements of tissue factor, predisposing patients to hypercoagulable events [18], and tissue ischemia. The complement and coagulation cascades are closely interlinked, substantially increasing patient risk for thrombus formation. Thrombin has been demonstrated to play a role in PNH via activation of the C3 and C5 components of the complement system. This increases the risk of thrombosis via a positive feedback loop where thrombin increases complement-mediated hemolysis leading to a cycle of hypercoagulability [7]. In this case, recurrent PNH flares due to sepsis likely led to recurrent hypercoagulability, recurrent splenic vascular ischemia, and eventual AS. Treatment with eculizumab significantly reduces the risk of thrombosis and improves survival in patients with PNH [19]. The delay in treatment initiation despite a large PNH clone population, progressive symptoms, and significant anemia secondary to patient refusal likely contributed to this patient's resultant AS, progressive decline in renal function, and development of deep venous thrombosis.

Eculizumab inhibits terminal complement activity by binding to C5 which is an important mechanism of defense against *Neisseria species*, thus vaccination is recommended [20–22]. Infections with other encapsulated organisms including *Haemophilus influenza* type B and *Streptococcus pneumoniae* are typically not associated with C5 or the alternative complement pathway but rather with C3 deficiency and deficiencies of components in the classical pathway [20, 21]. The spleen plays a crucial role in macrophage-mediated destruction of opsonized encapsulated bacteria, so splenic damage can also predispose patients to overwhelming infections by encapsulated organisms [21]. It also has roles in both the adaptive and innate arms of the immune system and serves as a filter for other hematogenous pathogens and antigens [23]. Due to the possibility of AS arising from PNH, the predisposition to infection by encapsulated organisms must be considered. This case indicates the possibility of splenic damage early in the presentation of PNH, so the presence of Howell–Jolly bodies on peripheral blood smear or recurrent infections may indicate the need for a workup to assess splenic size and function.

## 4. Conclusion

This is the first known case of autosplenectomy in a patient with PNH. This was probably due to a recurrent hypercoagulable environment from untreated PNH leading to repeated episodes of splenic ischemia, infarction, and eventual atrophy with autosplenectomy. This case demonstrates that splenic damage may present early in PNH onset, and presence of Howell–Jolly bodies or recurrent infections should prompt consideration of splenic imaging, especially given the propensity of PNH to manifest with thrombi in unusual locations.

## Abbreviations

AS: Autosplenectomy
PNH: Paroxysmal nocturnal hemoglobinuria
MCD: Minimal change disease
ESRD: End-stage renal disease
SCA: Sickle cell anemia.
